# Effects of escitalopram therapy on resting-state functional connectivity of subsystems of the default mode network in unmedicated patients with major depressive disorder

**DOI:** 10.1038/s41398-021-01754-4

**Published:** 2021-12-13

**Authors:** Jian Cui, Yun Wang, Rui Liu, Xiongying Chen, Zhifang Zhang, Yuan Feng, Jingjing Zhou, Yuan Zhou, Gang Wang

**Affiliations:** 1 The National Clinical Research Center for Mental Disorders & Beijing Key Laboratory of Mental Disorders, Beijing Anding Hospital, Capital Medical University, Beijing, 100088 China; 2 Advanced Innovation Center for Human Brain Protection, Capital Medical University, Beijing, 100069 China; 3 CAS Key Laboratory of Behavioral Science, Institute of Psychology, Beijing, 100101 China; 4 Department of Psychology, University of Chinese Academy of Sciences, Beijing, 100049 China

**Keywords:** Predictive markers, Depression

## Abstract

Antidepressants are often the first-line medications prescribed for patients with major depressive disorder (MDD). Given the critical role of the default mode network (DMN) in the physiopathology of MDD, the current study aimed to investigate the effects of antidepressants on the resting-state functional connectivity (rsFC) within and between the DMN subsystems. We collected resting-state functional magnetic resonance imaging (rs-fMRI) data from 36 unmedicated MDD patients at baseline and after escitalopram treatment for 12 weeks. The rs-fMRI data were also collected from 61 matched healthy controls at the time point with the same interval. Then, we decomposed the DMN into three subsystems based on a template from previous studies and computed the rsFC within and between the three subsystems. Finally, repeated measures analysis of covariance was conducted to identify the main effect of group and time and their interaction effect. We found that the significantly reduced within-subsystem rsFC in the DMN core subsystem in patients with MDD at baseline was increased after escitalopram treatment and became comparable with that in the healthy controls, whereas the reduced within-subsystem rsFC persisted in the DMN dorsal medial prefrontal cortex (dMPFC) and medial temporal subsystems in patients with MDD following escitalopram treatment. In addition, the reduced between-subsystem rsFC between the core and dMPFC subsystem showed a similar trend of change after treatment in patients with MDD. Moreover, our main results were confirmed using the DMN regions from another brain atlas. In the current study, we found different effects of escitalopram on the rsFC of the DMN subsystems. These findings deepened our understanding of the neuronal basis of antidepressants’ effect on brain function in patients with MDD. The trial name: appropriate technology study of MDD diagnosis and treatment based on objective indicators and measurement. URL: http://www.chictr.org.cn/showproj.aspx?proj=21377. Registration number: ChiCTR-OOC-17012566.

## Introduction

Major depressive disorder (MDD) has a high lifetime prevalence nearly up to 20.6% in adults [1], and MDD is regarded as the third non-fatal leading cause of the global burden of disease [2]. Antidepressant therapy (i.e., selective 5-HT reuptake inhibitors [SSRIs]) is the first-line treatment for patients with MDD [3]. Although it is well-known that most antidepressant medications primarily modulate monoaminergic neurotransmitters (such as serotonin [5-HT]), translation of these neurobiological changes into clinically important events remains unclear [4]. Neuroimaging studies in the last decades have found abnormal communications among large-scale brain networks, including the default mode network (DMN), frontoparietal network, and other networks related to emotion or salience processing in patients with MDD [5, 6]. Thus, identifying the effects of antidepressants on the brain networks is important to elucidate the neurobiological mechanisms of antidepressant action and develop targets for new interventions.

Among the brain networks related to MDD, the DMN draws increasing researchers’ attention in MDD studies. Evidence suggests that the DMN is responsible for self-referential processing [7, 8], and this function is impaired in patients with MDD, such as disordered self-referential thought and maladaptive rumination [9–12]. Using resting-state functional connectivity (rsFC), which detects synchronized spontaneous activity across anatomically distinct brain regions [13], previous functional magnetic resonance imaging (fMRI) studies have frequently reported abnormal rsFC within the DMN in patients with MDD. The seminal study conducted by Greicius and his colleagues has shown increased rsFC of the subgenual cingulate and thalamus with the DMN in the depressed subjects [14]. The increased rsFC within the DMN in patients with MDD is supported by a recent meta-analysis study [6]. However, decreased rsFC within the DMN has also been reported in patients with MDD. For example, based on the dataset consisting of 1300 patients with MDD and 1128 healthy controls (HCs) from 25 sites of China, Yan et al. [15] found decreased rsFC within the DMN in patients with MDD.

The DMN may be a vital target of treatment response of antidepressant medications at the neural circuit level because its functionality is modulated by several neurotransmitter systems or their interactions, such as serotonin, dopamine, and gamma-aminobutyric acid [16–18], which are either neurobiological targets of antidepressant medications or implicated in MDD pathophysiology [19–21]. Among these, the relationship between DMN and serotonin has attracted our attention. First, the expression of several 5-HT receptors spatially overlaps with the main DMN regions [22]. A study combining positron-emission tomography and fMRI showed that the rsFC of the DMN can be predicted by individual variations in the 5-HT 1A receptor binding in the dorsal raphe nucleus and in the project regions [16]. Second, pharmacologic neuroimaging studies indicated that the functional connectivity of the DMN is modulated by serotonin levels. For example, depletions of the 5-HT precursor tryptophan can change the rsFC of the DMN [23–25]. SSRIs, which can block the reuptake of 5-HT by inhibiting the 5-HT transporter (SERT), may decrease DMN connectivity [26, 27]. Furthermore, the modulation of the serotonin system on the DMN is dose-dependent as indicated by the occupancy level of SERT by citalopram (one of the SSRIs) [28]. All these studies suggest that the DMN is an important network in understanding the underlying mechanism of the antidepressant treatment of MDD, especially SSRIs. Therefore, the current work focused on the DMN and attempted to investigate the effect of SSRIs on the rsFC of the DMN.

Notably, the DMN can be divided into three subsystems [8], that is, the midline core subsystem, dorsal medial prefrontal cortex (dMPFC) subsystem, and medial temporal lobe (MTL) subsystem. The core subsystem, including the anterior medial prefrontal cortex (aMPFC) and posterior cingulate cortex (PCC), participates in the processes of self-related activity regardless of temporal context and links all of the three subsystems [29]. The dMPFC subsystem, comprising the dMPFC, temporal poles, lateral temporal cortex, and temporoparietal junction, is predominantly involved in meta-cognitive processes and mentalizing [29]. The MTL subsystem, comprising the hippocampal formation, retrosplenial cortex, and inferior parietal lobule, is associated with recollection of experiences and autobiographical processing [29]. The subsystems may be differently affected by the antidepressant medications because of their heterogeneity in functions. On the one hand, the modulation role of the 5-HT neurotransmitter system on the rsFC of the DMN is identified in the regions within the core subsystem [16]. On the other hand, previous fMRI studies have often reported altered local spontaneous neuronal activities and rsFC in the DMN core subsystem in patients with MDD following acute-phase SSRI treatment [30, 31]. For example, one study has found that escitalopram treatment for 4 weeks induces an increase of local spontaneous brain activity in the MPFC and middle cingulate cortex [30]. Another study has found that treatment response is associated with decreased rsFC between amygdala and the right precuneus as well as the right PCC after antidepressant treatment (including fluoxetine and sertraline) [31]. Therefore, the subsystems of the DMN may be affected by antidepressants to a different extent, and the core subsystem is more likely affected by antidepressants in patients with MDD. Based on previous reports, only one study has noted the dissociation effect of antidepressants on the DMN and found that after 12 weeks of antidepressant treatment, rsFC within the posterior component of the DMN was changed to a level similar to that seen in HCs, whereas rsFC within the anterior component persisted in patients with MDD [32]. However, in this study, the DMN was divided into two subsystems, with the MPFC in the anterior subsystem and the bilateral precuneus in the posterior subsystem. Moreover, in this study, patients were treated with several antidepressant medications, including SSRIs and serotonin–norepinephrine reuptake inhibitors (SNRIs), which might generate different effects on the brain circuits [33]. Therefore, it is still unclear on the effect of antidepressant treatment on the rsFC of the DMN subsystems in patients with MDD.

In the current study, we selected escitalopram, a highly selective serotonin reuptake inhibitor, as the single antidepressant medication and investigated the effect of 12-week antidepressant treatment on the rsFC of the 3 DMN subsystems. We are particularly interested in the rsFC within and between each subsystem of the DMN. Notably, we recruited a group of unmedicated patients with MDD to exclude the confounding effect of previously prescribed medications. The same as our patients matched HCs were also scanned at baseline and after 12 weeks. Such a strategy can exclude confounders of test–retest noise and provide a reliable reference to identify whether brain alterations persist in patients with MDD after treatment with escitalopram [34]. We hypothesize that the rsFC within or related to the core subsystem might be particularly influenced by the treatment of escitalopram because of its functionality and the modulation effect observed in previous studies. Moreover, we hypothesize that not all of the rsFC within the DMN subsystems or between-subsystems after treatment will be changed to a level similar to that seen in HCs, and some abnormalities in the rsFCs might persist in patients after treatment based on previous studies [32, 35].

## Materials and methods

### Participants

This study was conducted in Beijing Anding Hospital, Capital Medical University, an affiliated teaching hospital in Beijing, China. We recruited 40 unmedicated patients with MDD from the outpatient departments. The diagnosis of patients with MDD was made by trained psychiatrists using the Mini International Neuropsychiatric Interview (MINI) 5.0 [36] based on the DSM-IV criteria at the entry of this study. The inclusion criteria of the patients were as follows: male or female outpatients aged at least 18 years and not more than 65 years; systemic anti-depressants treatment were not adopted during a current episode or has taken anti-depressants less than 7 days in last 14 days; total score of the 16-Item Quick Inventory of Depressive Symptomatology and Self-Report (QIDS-SR16) ≥ 11 [37] and score of the Chinese version of the 17-item Hamilton Depression Rating Scale (HAMD-17) ≥ 14 [38] at enrollment in open-label preliminary phase; at least primary school education and understand the scales; and preparing to use escitalopram. The exclusion criteria of patients included history of manic episode or hypomanic episode; history of bipolar, schizophrenia, schizoaffective disorder, or other psychotic disorders; history of drug and alcohol dependence or acute intoxication; women in pregnancy or lactation; significant risk of suicidal behaviors; HAMD-17 Item 3(suicide) score ≥ 3; current clinically significant disease; previously intolerant or lack of response to escitalopram and any MRI contraindications.

HCs (*N* = 64) were recruited by advertisements and were interviewed by using the MINI to exclude any DSM-IV Axis I diagnosis. All healthy participants met the same additional exclusion criteria as the patients with MDD and were matched on age, gender composition and educational level with the patients with MDD. All participants signed informed consent to participate in the current study. This study was approved by the Ethics Committee of Beijing Anding Hospital, Capital Medical University.

### Treatment and measurements

All patients with MDD were treated with escitalopram for 12 weeks. Head-to-head studies have confirmed that escitalopram is one of the antidepressants with better efficacy and acceptability than others [39, 40]. In Asian countries, escitalopram is one of the most frequently prescribed drugs in patients with MDD [41, 42]. In accordance with the Clinical Practice Guidelines, the dose of escitalopram increased from 5 mg/day to 10–20 mg/day within 7 days, and the dose remained unchanged until the patients completed the 12-week study [43]. Patients experiencing insomnia symptoms were permitted to receive additional medications as needed. No other medications were permitted to be used during the study. Finally, 13 patients received additional medications, including estazolam for 2 patients, lorazepam for 9 patients, and oxazepam for 2 patients. The HAMD-17 was assessed by trained and experienced independent raters. All patients were evaluated at baseline and after 12 weeks. All participants also completed the self-reported questionnaire of Patient Health Questionnaire-9 (PHQ-9) [44].

Seven participants, including four MDD patients and three HCs, were excluded. Four MDD patients were excluded because of non-adherence to the protocol (*N* = 1) or excessive head movement (*N* = 3, see details in the following section). Three HCs were excluded because of non-adherence to the protocol during the follow-up period. A total of 97 participants were included in the final analysis, of which 36 were MDD patients and 61 were HCs. The demographic characteristics of the participants are shown in Table 1.

**Table 1:** Demographics and clinical characteristics.

	MDD (*N* = 36)	HCs (*N* = 61)	*P*
Age (years, mean ± SD)	27.5 ± 5.88	26.16 ± 4.38	0.21^a^
Age (years, range)	18–46	19–40	
Gender (male/female)	11/25	22/39	0.66^b^
Education (H/U/G)	5/24/7	4/40/17	0.37^b^
Number of previous episodes	1.64 ± 1.29	–	–
Baseline headmotion (mean FD)	0.16 ± 0.07	0.13 ± 0.07	0.74^c^
Follow-up headmotion (mean FD)	0.15 ± 0.09	0.14 ± 0.09	
Baseline PHQ-9	17.58 ± 4.8	2.23 ± 1.9	<0.001^c^
Follow-up PHQ-9	6.31 ± 5.71	1.41 ± 1.4	
Baseline HAMD-17	21.86 ± 3.25	–	<0.001^a^
Follow-up HAMD-17	8.11 ± 5.04	–	

Notes: Values are shown in mean ± SD. Abbreviations: *MDD* major depressive disorder, *HCs* healthy controls, *FD* framewise displacement, *PHQ-9* Patient Health Questionnaire-9, *HAMD-17* 17-item Hamilton Depression Rating Scale, *H* high school, *U* undergraduate, *G* graduate.

^a^Age and HAMD scores were analyzed by using the two-sample *t* test.

^b^Gender and education were analyzed by using the chi-square test.

^c^Headmotion and PHQ-9 were analyzed by using two-way repeated analysis of covariance.

### MRI data acquisition

All scans were performed by using a 3.0 T Siemens MAGNETOM Prisma MRI scanner (Siemens Medical Solutions, Erlangen, Germany) with a 64-channel phased-array head coil. Foam paddings were used to minimize head movement, and earplugs were used to minimize scanner noise. A total of 200 volumes of functional images were obtained axially with an echo-planar imaging sequence: number of slices = 33, repetition time (TR) = 2000 ms, echo time (TE) = 30 ms, flip angle = 90°, field of view (FOV) = 200 × 200 mm^2^, phase encoding direction = anterior to posterior, in-plane matrix resolution = 64 × 64, slice thickness = 3.5 mm, gap = 0.7 mm and voxel size = 3.13 × 3.13 × 4.2 mm^3^. High-resolution sagittal T1 images were acquired by using the 3D magnetization-prepared rapid gradient-echo sequence: TR = 2530 ms, TE = 1.85 ms, flip angle = 15°, FOV = 256 × 256 mm^2^, slices number = 192, with a thickness of 1 mm, no gap, voxel size = 1 × 1 × 1 mm^3^. Before scanning, participants were instructed to keep awake with their eyes closed, not to think any particular thing, and try their best to keep still without any head motion. The duration of the resting-state fMRI scanning was 6 min and 40 s. The patients with MDD underwent identical MRI scan sequences at baseline and after treatment for 12 weeks. The HCs also experienced two scans with the same interval.

### Resting-state fMRI data preprocessing

Data preprocessing steps were performed by using a data processing assistant for resting-state fMRI (DPARSF_V4.5, http://rfmri.org/DPARSF) [45]. First, we removed the first five-time points to exclude possible magnetization effects. Then, we performed slice timing; realignment; segmentation of T1 structural images to generate gray matter, white matter (WM), and cerebrospinal fluid (CSF); nuisance covariate regression; normalization to MNI space (voxel size = 2 × 2 × 2 mm^3^), spatial smoothing with a 4 mm FWHM kernel, and band-pass filtering (0.01–0.1 Hz). The nuisance covariates included linear and quadratic trends, the first five principal components of the individually segmented WM and CSF, and Friston’s 24 motion parameters (six head motion parameters, six head motion parameters one-time point before, and 12 corresponding squared items) [46, 47]. We did not regress out the global signals in the nuisance covariates regression because doing so may lead to artificial negative correlations in rsFC analysis [48] and distort between-group effects [49, 50].

We limited our data analyses to participants with a receivable range of head motion to reduce the effect of motion-related artifacts on rsFC. First, we used volume-based Frame-wise Displacement (FD) to quantify micro-head motions [51], and participants who had less than 100 “good” volumes of data (a threshold of FD ≤ 0.5 mm) were excluded [52]. Second, participants with severe head motion (above three standard deviations of mean FD beyond the mean value) were excluded. Collectively, we excluded three patients. Moreover, we employed volume-based scrubbing regression by including scrubbing regressors as nuisance covariates [52], and we used the mean FD as a covariate in group-level analyses.

### Definition of regions of interest (ROIs) within the DMN subsystems

**Fig. 1:**
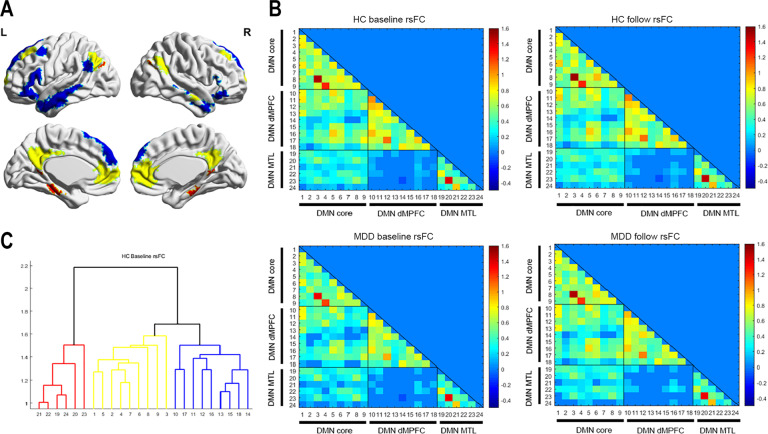
Default mode network subsystems. Panel **A** shows spatial distributions of the three subsystems of the default mode network from Yeo’s template [54, 55]. Brain regions painted in yellow belong to the core subsystem; brain regions painted in blue belong to the dMPFC subsystem, and brain regions painted in red belong to the MTL subsystem. Panels of **B** show the lower triangular of the averaged functional connectivity matrices among 24 ROIs of the default mode network in the healthy controls and the patients with MDD at baseline and after 12 weeks. The color bar represents functional connectivity strength. Panel **C** shows the result of hierarchical clustering analysis for the connectivity matrix of the healthy controls at baseline, which were consistent with Yeo’s default mode subsystems. The meaning of the color of the number is the same as those in Panel A. See Table S2 for the meaning of numbers.

We used a total of 24 anatomical ROIs created by Yeo et al. [53] based on their 17-network parcellation, which was derived from data of 1000 young healthy participants [54, 55]. These 24 ROIs could be divided into three DMN subsystems: nine ROIs in the core subsystem, nine ROIs in the dMPFC subsystem, and six ROIs in the MTL subsystem [8, 53]. These ROIs are shown in Fig. 1A and Table S1.

### Functional connectivity analyses

The mean time series were extracted from each ROI. Pearson correlation coefficient between the mean time series of each pair of these 24 ROIs was computed, resulting in a 24 × 24 functional connectivity matrix for each participant. Then, Fisher r-to-z transformation was conducted for all rsFC values. On the basis of this normalized connectivity matrix of each participant, we computed the rsFC within and between the subsystems of the DMN [15, 56]. In particular, two types of network connectivity (within-subsystem and between-subsystem) for the three subsystems were computed on the basis of the connectivity matrices [57]: the within-subsystem connectivity for each subsystem was calculated as the averaged connectivity across all the links within the subsystem normalized by the square of the number of nodes (ROIs). The pairwise connectivity between subsystems was computed as the averaged connectivity across all the links between two subsystems normalized by the product of the number of nodes within each of the two subsystems.

### Statistical analysis

A two-sample *t* test, and chi-squared test were used to assess the differences in demographic data between the patients with MDD and the HCs using SPSS version 23.0. Hierarchical clustering analysis was performed to validate that these ROIs were grouped into three pre-defined subsystems in the HCs. Then, a two-way repeated-measures analysis of covariance was performed to determine the main effects of group (MDD patients vs. HCs), time (baseline vs. 12 weeks), and the group × time interaction on each within-subsystem rsFC and between-subsystem rsFC, with age, gender, educational level, and head motion (mean FD) as nuisance covariates. *P* values < 0.05 were adjusted for multiple comparisons by controlling the false-discovery rate (FDR).

We also conducted group-level analyses on the rsFC of each pair of these 24 ROIs to give a comprehensive view of the treatment-related effect on rsFC. We used Network-Based Statistics (NBS) [58] to identify sub-networks, in which the rsFC was affected by the main effect of group and time and their interaction effect. Based on our research aim, here we only focused on the interaction effect, that is, treatment-related effect. Analogous to cluster-based correction strategies used in voxel-wise fMRI studies, the NBS [58] focused on the multiple-comparison problem posed by connectomic data by evaluating the null hypothesis at the level of interconnected sub-networks rather than individual connections. To realize this analysis, we used the NBS toolbox (https://www.nitrc.org/) to analyze the functional matrices with statistical threshold: *t*-threshold = 5.5, 5000 permutations, and corrected *P* < 0.05.

Within-subsystem and between-subsystem rsFC values were extracted in the patient group to explore the relationship between changes of the DMN functional connectivity (rsFC_12W_–rsFC_0W_) and the clinical improvement (HAMD_0W_–HAMD_12W_). Pearson correlation analysis was conducted between the changes of rsFC values and the clinical improvement. The relationship between the DMN subsystem connectivity (within- and between-subsystem connectivity) at baseline and the clinical improvement was also explored. Owing to the small sample size, an uncorrected statistical significance level of *P* < 0.05 was used.

### Confirmation analyses

We repeated our analyses by using another functional brain atlas (Power 264 atlas, which included 58 DMN regions) to exclude the influence of potential variability in ROI selection on our results [59] (for details, see the supplementary materials). We intersected the 58 ROIs of DMN in the Power atlas [59] with Yeo’s DMN template to exclude 11 ROIs, which resulted in 47 ROIs for subsequent analyses. Then, within-subsystem and between-subsystem connectivity for the three subsystems were computed using the same methods for the main analyses.

In addition, the main analyses were repeated by comparing the responders and non-responders to escitalopram. The responder exhibited a more than 50% reduction in the initial HAMD-17 scores. The main analyses in the responders were also repeated by comparing the DMN subsystem connectivity between the responders and HCs. Finally, the DMN subsystem connectivity in the HCs between the two-time points was compared by paired *t* tests to determine the stability of the main findings.

## Results

### Demographic and clinical characteristics

The demographic characteristics of the subjects are shown in Table 1. Distributions of age, gender, educational level, and head motion (mean FD) were not significantly different among the patients with MDD and the HCs (all *P*-values > 0.05). The HAMD-17 total scores of the patients were significantly decreased after 12-week treatment (*P* < 0.05). Among the patients, 28 (77.8%) achieved response defined as a reduction of 50% or more in the HAMD-17 score, and 19 patients (52.8%) showed clinical remission with HAMD-17 score ≤ 7. The PHQ-9 total scores of patients with MDD were also significantly decreased after 12-week treatment (*P* < 0.05). Moreover, both of the scores of patients with MDD at baseline and after 12 weeks were significantly higher than those of the HCs (*P*-values < 0.05), in whom no difference in the PHQ-9 total scores was found at baseline and after 12 weeks (*P* > 0.05), and the scores of the HCs were all below 5.

### DMN subsystem connectivity within each group

The lower triangular of the averaged functional connectivity matrix was displayed for each group (Fig. 1B). The hierarchical clustering analysis verified that the DMN ROIs included in our studies were grouped into the predefined subsystems in the HCs, as reported in previous studies [56, 60] (Fig. 1C). The MDD patients showed similar connectivity patterns among the 24 ROIs as the HCs.

### Group × time interaction effect on the DMN subsystem connectivity

**Fig. 2:**
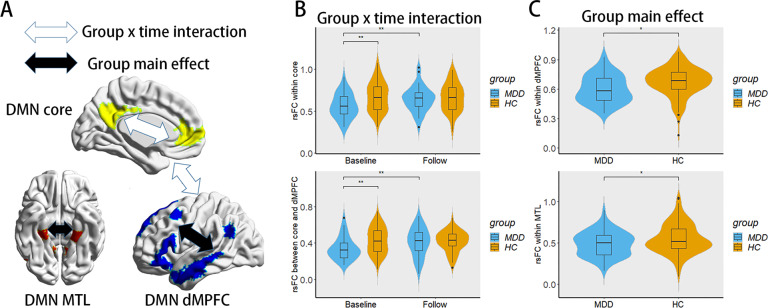
The interaction effect and main effect on the rsFC within and between the DMN subsystems. Panel **A** summarizes the interaction effect and main effect within and between the DMN subsystems. Panel **B** shows the significant interaction effect on the within-subsystem rsFC of the core subsystem and the between-subsystem rsFC of the core and dMPFC subsystem using violin plots. Panel **C** shows the significant group main effect on the within-subsystem rsFC in the dMPFC and MTL subsystems using violin plots. **P-values < 0.01; *P-values < 0.05 for post hoc analyses.

A significant interaction effect between group and time was found in the within-subsystem rsFC of the core subsystem (*P* < 0.05, FDR corrected; Fig. 2A). Post hoc analysis showed that within-subsystem rsFC values of the core subsystem in the MDD patients at baseline were significantly lower than those of the HCs at baseline (*P* = 0.007, Bonferroni corrected) and significantly lower than those of the MDD patients after treatment (*P* = 0.013, Bonferroni corrected); however, within-subsystem rsFC values of the core subsystem in the MDD patients after treatment were not significantly different from those of the HCs after 12 weeks (*P* > 0.05, Bonferroni corrected) (Fig. 2B). In addition, we found a trend toward the significance of the interaction effect on the rsFC between the core and dMPFC subsystem (*P* = 0.055, FDR corrected). Post hoc analysis showed that between-subsystem rsFC values of the core and dMPFC subsystems in the MDD patients at baseline were significantly lower than those of the HCs at baseline (*P* = 0.008, Bonferroni corrected) and significantly lower than those of the MDD patients after treatment (*P* = 0.013, Bonferroni corrected); however, between-subsystem rsFC values of the core and dMPFC subsystems in the MDD patients after treatment were not significantly lower than those of the HCs after 12 weeks (*P* > 0.05, Bonferroni corrected). The rsFC values (z transformed) of within and between-DMN subsystems are shown in Table 2.

**Table 2:** Significant effects of main and interaction on the rsFC (z transformed) within and between the DMN subsystems.

		MDD baseline (mean ± SD)	MDD week 12 (mean ± SD)	HCs baseline (mean ± SD)	HCs week 12 (mean ± SD)	*F* group (P)	*F* time (P)	*F* interaction (P)
Within subsystem	Core subsystem	0.57 ± 0.15	0.65 ± 0.15	0.68 ± 0.18	0.66 ± 0.17	3.63(0.06)	3.15(0.16)	6.19(0.04*)
	dMPFC subsystem	0.57 ± 0.13	0.63 ± 0.15	0.67 ± 0.17	0.68 ± 0.13	6.63(0.04*)	2.62(0.16)	2.25(0.21)
	MTL subsystem	0.49 ± 0.14	0.50 ± 0.16	0.53 ± 0.15	0.57 ± 0.20	5.00(0.04*)	1.77(0.19)	0.67(0.42)
Between subsystem	Core-dMPFC subsystem	0.34 ± 0.12	0.41 ± 0.16	0.43 ± 0.15	0.42 ± 0.11	3.41(0.20)	3.11(0.24)	5.77(0.05*)
	Core-MTL subsystem	0.24 ± 0.12	0.26 ± 0.14	0.28 ± 0.18	0.30 ± 0.16	1.20(0.41)	1.46(0.34)	0.00(0.96)
	dMPFC-MTL subsystem	0.05 ± 0.12	0.07 ± 0.14	0.05 ± 0.16	0.06 ± 0.14	0.05(0.82)	0.55(0.46)	0.25(0.93)

Notes: *P* values after FDR correction. *significant after FDR correction (*P* < 0.05). Abbreviations: *MDD* major depressive disorder, *HCs* healthy controls, *dMPFC* dorsal medial prefrontal cortex, *MTL* medial temporal lobe.

**Fig. 3:**
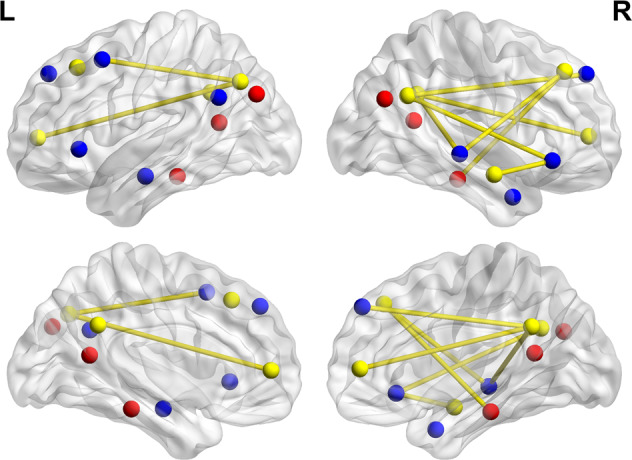
The interaction effect on the ROI-to-ROI rsFC. The ROI regions painted in yellow belong to the core subsystem; the ROI regions painted in blue belong to the dMPFC subsystem, and the ROI regions painted in red belong to the MTL subsystem.

For the rsFC of each pair of ROIs, we found that the group and time interaction effect was significant in 19 connections (*P* < 0.05, NBS corrected; Fig. 3 and Table 3). Similar to the findings obtained in the subsystem level, the interaction effect involved the connectivity within the core subsystem (7 connections) and between the core and the dMPFC subsystems (10 connections). Post hoc analysis showed that all of these ROI-to-ROI rsFC values in the MDD patients at baseline were significantly lower than those of the HCs at baseline (*P*-values < 0.05, Bonferroni corrected) and significantly lower than those of the MDD patients after treatment (*P*-values < 0.05, Bonferroni corrected); however, all of these rsFC values in the MDD patients after treatment were not significantly lower than those of HCs after 12 weeks (*P*-values > 0.05, Bonferroni corrected).

**Table 3:** Significant interaction effect on the ROI-to-ROI rsFC (z transformed).

Region	MNI coordinates			Region	MNI coordinates			MDD baseline (mean ± SD)	MDD week 12 (mean ± SD)	HCs baseline (mean ± SD)	HCs week 12 (mean ± SD)	*F* interaction (*P*)
	(*x*	*y*	*z*)		(*x*	*y*	*z*)					
*Within core subsystem*												
IPL.L	−44	−68	37	PFCm.L	−8	51	4	0.48 ± 0.23	0.54 ± 0.27	0.58 ± 0.22	0.52 ± 0.26	5.57 (0.02)
PFCm.L	−8	51	4	Temp.L	62	−5	−17	0.34 ± 0.27	0.54 ± 0.22	0.44 ± 0.31	0.46 ± 0.28	11.38 (<0.01)
PFCm.L	−8	51	4	IPL.L	51	−57	29	0.35 ± 0.26	0.47 ± 0.20	0.45 ± 0.24	0.42 ± 0.27	6.36 (0.01)
pCunPCC.L	−5	−51	31	PFCd.L	23	35	43	0.45 ± 0.22	0.55 ± 0.34	0.57 ± 0.21	0.52 ± 0.26	6.73 (0.01)
PFCm.L	−8	51	4	PFCd.L	23	35	43	0.42 ± 0.25	0.56 ± 0.29	0.54 ± 0.20	0.48 ± 0.26	12.12 (<0.01)
PFCm.L	−8	51	4	PFCl.L	7	−51	30	0.51 ± 0.23	0.65 ± 0.23	0.62 ± 0.25	0.61 ± 0.31	6.16 (0.01)
PFCl.L	7	−51	30	PFCv.L	7	49	5	0.62 ± 0.26	0.78 ± 0.26	0.74 ± 0.25	0.73 ± 0.29	6.54 (0.01)
*Core-dMPFC subsystem*												
Temp.L	62	−5	−17	IPL.L	−56	−12	−18	0.73 ± 0.22	0.86 ± 0.25	0.83 ± 0.27	0.81 ± 0.22	6.52 (0.01)
IPL.L	51	−57	29	PHC.L	−8	44	42	0.32 ± 0.21	0.48 ± 0.23	0.46 ± 0.25	0.48 ± 0.21	7.26 (0.01)
IPL.L	−44	−68	37	Temp.R	−40	13	50	0.63 ± 0.31	0.72 ± 0.33	0.76 ± 0.31	0.69 ± 0.28	6.50 (0.01)
Temp.L	62	−5	−17	Temp.R	−40	13	50	0.23 ± 0.22	0.36 ± 0.25	0.37 ± 0.29	0.35 ± 0.25	5.93 (0.02)
Temp.L	62	−5	−17	IPL.R	−47	26	−2	0.19 ± 0.22	0.31 ± 0.25	0.31 ± 0.25	0.28 ± 0.17	7.37 (0.01)
PFCd.L	23	35	43	PFCd.R	62	−26	−5	0.18 ± 0.25	0.29 ± 0.25	0.31 ± 0.25	0.25 ± 0.28	6.73 (0.01)
PFCl.L	7	−51	30	PFCd.R	62	−26	−5	0.30 ± 0.21	0.40 ± 0.23	0.45 ± 0.24	0.40 ± 0.22	8.20 (0.01)
IPL.L	51	−57	29	PFCm.R	10	48	41	0.55 ± 0.23	0.66 ± 0.25	0.68 ± 0.30	0.67 ± 0.21	5.71 (0.02)
Temp.L	62	−5	−17	Temp.R	46	28	−9	0.28 ± 0.25	0.44 ± 0.22	0.38 ± 0.25	0.40 ± 0.23	5.56 (0.02)
IPL.L	51	−57	29	Temp.R	46	28	−9	0.27 ± 0.24	0.41 ± 0.25	0.35 ± 0.23	0.34 ± 0.22	7.45 (0.01)
*dMPFC-MTL subsystem*												
IPL.L	−56	−12	−18	Temp.R	46	28	−9	0.40 ± 0.22	0.56 ± 0.28	0.50 ± 0.27	0.51 ± 0.22	5.82 (0.02)
*Core-MTL subsystem*												
PFCd.L	23	35	43	PHC.R	26	−27	−20	0.15 ± 0.17	0.18 ± 0.20	0.08 ± 0.20	0.23 ± 0.21	6.79 (0.01)

Notes: *P* values after NBS correction (only list the connections with *P* < 0.05). Abbreviations: *L* left, *R* right, *IPL* inferior parietal lobule, *pCunPCC* precuneus posterior cingulate cortex, *PFCd* dorsal prefrontal cortex, *PFCl* lateral prefrontal cortex, *PFCm* medial prefrontal cortex, *PFCv* ventral prefrontal cortex, *PHC* parahippocampal cortex, *Temp* temporal lobe.

### The main effect of group and time on DMN subsystem connectivity

We found a significant effect of group on within-subsystem rsFC in the dMPFC and MTL subsystems (*P* < 0.05, FDR corrected). As shown in Fig. 2C, within-subsystem rsFC values of the dMPFC or the MTL subsystem in the MDD patients were significantly lower than those of the HCs (*P* < 0.05, FDR corrected). No main effect of group was found in other within- or between-subsystem rsFC. In addition, no main effect of time was found.

### Correlations between the DMN subsystem connectivity and clinical improvements

No significant correlations between the changes of within-subsystem and between-subsystem rsFC values and the clinical improvement were observed among the patients with MDD (*P*-values > 0.05, uncorrected). However, a significant negative correlation was found between the rsFC within the MTL subsystem at baseline and the clinical improvement, suggesting that the low rsFC within the MTL subsystem at baseline was associated with good clinical improvement (*r* = −0.33, *P* = 0.05, uncorrected).

### Confirmation analyses

In the confirmation analysis using the DMN regions from the Power 264 atlas as the 47 ROIs, the interaction effect showed a trend toward significance in the within-subsystem rsFC of the core subsystem (*P* = 0.07, FDR corrected) and the between-subsystem rsFC between the core and dMPFC subsystems (*P* = 0.06, FDR corrected). Post hoc analysis showed that the reduced within-subsystem and between-subsystem rsFC in the patients with MDD at baseline was also increased following treatment (*P*-values < 0.05, Bonferroni corrected) and became comparable with those in the HCs. In addition, we found a significant group effect on the within-subsystem rsFC in the dMPFC subsystem (*P* < 0.05, FDR corrected). Thus, our main results were confirmed, except for the group effect on the MTL within-subsystem. Details could be found in the supplement materials (Fig. S1). No significant correlations were observed between the changes of within-subsystem and between-subsystem rsFC values and the clinical improvement (*P*-values > 0.05, uncorrected). The correlation between the rsFC within the MTL subsystem at baseline and the clinical improvement disappeared, and no correlations between the other DMN subsystem connectivity at baseline and the clinical improvement were observed in the confirmation analysis (*P*-values > 0.05, uncorrected).

Among patients completing the study, 28 were responders and 8 were non-responders. A two-way repeated-measure ANOVA was used to determine the main effects of subgroup (the responders vs. the non-responders), time (baseline vs. 12 weeks), and subgroup × time interaction on each within-subsystem rsFC and between-subsystem rsFC, with age, gender, educational level, and head motion (mean FD) as nuisance covariates. The significant main effect of subgroup or interaction effect was not observed on the DMN subsystem connectivity (*P*-values > 0.05, FDR corrected) (Table S2). However, the significant main effect of time on rsFC was noted between the core and dMPFC subsystem (*P* = 0.048, FDR corrected). A trend toward the significance of the main effect of time was also noted in the within-subsystem rsFC of the core subsystem (*P* = 0.051, FDR corrected). The main effect of time is consistent with our main finding, suggesting that rsFC related to the core subsystem increased after escitalopram treatment across patients with MDD.

Furthermore, the main analyses were confirmed in patients with MDD who responded to escitalopram. Similar to the main findings, an interaction effect between group and time in the within-subsystem rsFC of the core subsystem (*P* < 0.05, uncorrected) and a trend towards significance in the interaction effect of the rsFC between the core subsystem and the dMPFC subsystem (*P* = 0.07, uncorrected) were observed. The significant main effect of group on within-subsystem rsFC in the dMPFC subsystem and the MTL subsystem was also noted (*P* < 0.05, uncorrected). However, due to the small sample size, these effects observed in the responders cannot survive from the multiple comparison corrections (*P*-values > 0.05, FDR corrected). No main effect of group or time was found in other within- or between-subsystem rsFC. Details can be found in the supplement materials (Fig. S2).

Finally, no significant differences were found in within-subsystem rsFC or in the between-subsystem rsFC between at baseline and after the 12-week interval in the HCs (*P*-values > 0.05, FDR corrected) (Table S3). This result supports the stability of the main findings.

## Discussion

Our study investigated the effects of escitalopram on the rsFC of the three DMN subsystems in patients with MDD. We observed that after escitalopram treatment for 12 weeks, decreased within-subsystem rsFC of the DMN core subsystem in patients with MDD was increased to a level similar to that seen in HCs. The same trend was found in the between-subsystem rsFC between the core and dMPFC subsystems at a looser threshold. We also found that the decreased within-subsystem rsFC persisted in the dMPFC and MTL subsystems after treatment. Moreover, our main results were confirmed using the DMN regions from another brain atlas.

The main finding is that the rsFC related to the DMN core subsystem (rsFC within this subsystem and rsFC between the core and dMPFC subsystem) in the patients with MDD decreased at baseline and then became comparable with that seen in HCs after escitalopram treatment for 12 weeks. The core subsystem, composed of the aMPFC and PCC as key nodes, is commonly regarded as a key network in patients with MDD due to its role in self-referential processing [7, 11, 12]. Our finding supports DMN hypoconnectivity in patients with MDD at baseline. This finding seems to contradict previous reports, in which DMN hyperconnectivity was found in patients with MDD who have impaired self-referential processes, such as self-referential thought and maladaptive rumination [9, 10]. However, after an exhaustive search on literature, researchers found that less than 50% of studies (*N* = 18) reported hyperconnectivity, 21% (*N* = 8) reported hypoconnectivity, 18% (*N* = 7) reported increased and decreased connectivity, and 13% (*N* = 5) reported no significant changes in the DMN of patients with MDD (38 reports) (for a review, please see also [15]). Furthermore, a voxel-wise meta-analysis showed increased functional connectivity in the orbitofrontal DMN (including subgenual cingulate cortex) and decreased functional connectivity in the dMPFC and posterior DMN in patients with MDD [15]. Moreover, the decreased rsFC related to the DMN core subsystem at baseline in the current work was generally consistent with the finding obtained by the largest MDD database of China [15], in which the rsFC in the DMN was reduced in recurrent patients with MDD who had antidepressant treatments. The decreased rsFC between the core and dMPFC subsystems in the patients with MDD at baseline was also consistent with a previous study that recruited drug-naïve patients with MDD in the first episode [56]. The inconsistency between our findings and previous studies on hyperconnectivity or the mixed findings in the DMN may be attributed to the heterogeneity in MDD. Based on the largest MDD database of China, researchers found that the DMN connectivity was not associated with illness duration and showed no significant differences among clinical sybtypes [15]. The biotypes of MDD may account for the inconsistency across studies. The brain connectivity-based subtypes of MDD have been recently identified [61–63]. In particular, a study found decreased and increased DMN connectivity simultaneously occurring in patients with MDD, regardless of having the first episode. This finding suggests that the two biotypes of MDD exist, though no significant differences in demographic and clinical variables were found between patient subgroups [64]. The current study cannot parse the biotypes of MDD due to the small sample size; however, our findings provide new evidence for the hypoconnectivity of the DMN in patients with MDD. It is possible that our samples and others in previous studies [65–72] may be occasionally constituted of patients whose rsFC related to the DMN at baseline decreased. Additional studies must recruit more patients and parse the biotypes of MDD to validate our findings.

It should be noted that the rsFC related to the core subsystem in the patients with MDD were increased after escitalopram treatment for 12 weeks and became comparable with those of the HCs in the current study. This finding is compatible with two previous studies. In one study, depressed elderly participants had significantly higher functional connectivity between the PCC and the MPFC after treatment relative to that before treatment; however, the significance disappeared after adjusting for WM hyperintensity burden [73]. In another study, relative to placebo, acute citalopram administration increased rsFC between the PCC and MPFC [74]. The 5-HT neurotransmitter system primarily modulated the regions within the core subsystem; however, the core subsystem had widely anatomical and functional connections with the dMPFC subsystem [16, 75]. Therefore, in the current study, the change of the rsFC related to the core subsystem (rsFC within this subsystem and rsFC between the core and dMPFC subsystem) might indicate that escitalopram increases rsFC within the core subsystem by blocking the reuptake of the 5-HT and this effect extends to the rsFC between the core and dMPFC subsystem. The core subsystem is considered to integrate external or internal information with one’s prior episodic knowledge and current affective experience [76]. The dMPFC subsystem is primarily active when participants are making affective self-referential cognition, which is correlated with the core subsystem [77]. Thus, in the current study, the decreased rsFC related to the core subsystem in the patients with MDD at baseline might reflect the abnormalities in self-related processes in these patients and these abnormalities could be reversed by escitalopram treatment. This speculation can be supported by previous studies. Both of the decreased emotional response or brain activity to positive autobiographical memories and increased emotional response or brain activity to negative autobiographical memories are observed in the patients with MDD [78, 79]. More importantly, the decreased brain activity in the amygdala to positive autobiographical memories in the SSRI responders after treatment was changed to a level similar to that seen in HCs [80]. Future studies can use measurements on autobiographical memories or other self-related processes to test this speculation. In brief, our finding on the rsFC related to the core subsystem might be important to elucidate the neurobiological mechanisms of escitalopram action in patients with MDD.

In the current study, the decreased within-subsystem rsFC in the dMPFC and MTL subsystems persisted in the patients with MDD after treatment. Decreased neurochemical changes within the dMPFC subsystem and decreased rsFC within the MTL subsystem have been reported in previous studies [81, 82]. However, 12-week treatment of escitalopram did not change the decreased within-subsystem rsFC in the dMPFC and MTL subsystems in our study, which indicated that the decreased within-subsystem rsFC in the two subsystems might be independent of the presence of escitalopram therapy and thus might reflect disease-specific features of MDD. Therefore, these observations imply that the hypoconnectivity within the dMPFC and MTL subsystems may not be modulated by escitalopram and thus may reflect the abnormal neural circuit implicated in the pathophysiology of MDD. It is possible that the persistent abnormal functional connectivity within the dMPFC and MTL subsystems following treatment in patients with MDD may indicate a biomarker of diagnosis of MDD. However, due to the limited follow-up period (12 weeks), the possibility that the rsFC will be changed to a level similar to that seen in HCs after long-term treatment (e.g., 6 months or longer) cannot be excluded. Given the heterogeneity of MDD, future studies should recruit a large sample size and conduct a longitudinal design with a long follow-up period to validate the current findings.

Moreover, our main results were confirmed by using the DMN regions from the Power 264 atlas, except for the group effect on within-subsystem rsFC in the MTL subsystem or the clinical correlates of this subsystem. The unstable finding in the MTL subsystem might be due to the difference between the two atlases. The ROIs obtained from the Power atlas were spheres with 5 mm radius, but those in Yeo’s template were clusters covering the whole ROI. The difference in ROIs might affect the estimation of rsFC, which has been repeatedly discussed in previous studies [83, 84]. In the future, we hope to recruit other suitable templates to verify whether the group main effect in the MTL subsystem can be observed.

Insignificant correlations were found between the changes of the DMN subsystem connectivity and the clinical improvement after the 12-week treatment of escitalopram. The correlation between the changes of rsFC and clinical improvement in depression severity, which suggests a match between symptomatic recovery and MDD neurobiology, must be further explored [32]. However, only a few studies on the antidepressant’s effect on the rsFC have reported this correlation [31, 85–88], other works did not find [32, 89, 90] or did not report this correlation [73, 91]. The inconsistent findings combined with the small sample size across these studies (*N* = 12–21) appeal to future research with a large sample size to build a robust relationship between rsFC and clinical improvement.

The present study has several limitations. First, although patients who completed the study included responders (*N* = 28) and non-responders (*N* = 8) after the 12-week treatment of escitalopram, no difference was found between the responders and non-responders. Future studies must recruit additional MDD samples to find differences in the rsFC of the DMN between the responders and non-responders and verify the current results. Second, a placebo effect cannot be ruled out because we did not include a single group of MDD patients taking a placebo in the current study. The optimized controls would be groups of placebo-used or unmedicated patients with MDD. However, ethically speaking, asking patients with MDD who were experiencing depressive symptoms to remain untreated is a challenge. Therefore, recruiting patients with moderate depressive symptoms using a placebo for a short term could be helpful to confirm drug-specific effects in the future. Third, recent studies have shown that regions defined in the group-level atlas are suboptimal compared to individually specified regions in connectivity analyses [92, 93]. Therefore, future work could use the individual-level functional mapping which may better identify the variations in the effect of antidepressant treatment on brain functions in patients with MDD. Finally, the state of mind was not evaluated during the resting-state scanning. Future studies should use inventories to quantify the resting-state cognition, including sleepiness, comfort, and discontinuity of mind [94] to further understand the functional implications of the DMN subsystem connectivity.

## Conclusions

Our findings suggest that the DMN core subsystem may be a key DMN subsystem that plays an important role in the effect of escitalopram on brain functions for patients with MDD. We also found that the decreased within-subsystem rsFC in the dMPFC and MTL subsystems persisted in patients with MDD after treatment, indicating that the abnormality is independent of the presence of escitalopram therapy in MDD. The dissociation effect of escitalopram on the rsFC of DMN subnetworks deepens our understanding of the neural basis of antidepressants in patients with MDD. In addition, the persistent abnormal functional connectivity within the dMPFC and MTL subsystems following treatment in these patients may indicate a biomarker of diagnosis of MDD.

## Supplementary information

SUPPLEMENTAL MATERIAL

## Data Availability

The data that support the findings of this study are available from the corresponding authors upon reasonable request.

## References

[1] Hasin DS, Sarvet AL, Meyers JL, Saha TD, Ruan WJ, Stohl M (2018). Epidemiology of adult DSM-5 major depressive disorder and its specifiers in the United States. JAMA Psychiat.

[2] Spencer L James DAKH (2018). Global, regional, and national incidence, prevalence, and years lived with disability for 354 diseases and injuries for 195 countries and territories, 1990-2017: a systematic analysis for the Global Burden of Disease Study 2017. Lancet.

[3] APA, Practice Guideline for the Treatment of Patients with Major Depressive Disorder (3rd Edition). American Psychiatric Association (2000). 10767867

[4] Hirschfeld RM (2012). The epidemiology of depression and the evolution of treatment. J Clin Psychiatry.

[5] Williams LM (2017). Defining biotypes for depression and anxiety based on large-scale circuit dysfunction: a theoretical review of the evidence and future directions for clinical translation. Depress Anxiety.

[6] Kaiser RH, Andrews-Hanna JR, Wager TD, Pizzagalli DA (2015). Large-scale network dysfunction in major depressive disorder: a meta-analysis of resting-state functional connectivity. JAMA Psychiatr.

[7] Raichle ME, MacLeod AM, Snyder AZ, Powers WJ, Gusnard DA, Shulman GL (2001). A default mode of brain function. Proc Natl Acad Sci USA.

[8] Andrews-Hanna JR, Reidler JS, Sepulcre J, Poulin R, Buckner RL (2010). Functional-anatomic fractionation of the brain’s default network. Neuron.

[9] Sheline YI, Barch DM, Price JL, Rundle MM, Vaishnavi SN, Snyder AZ (2009). The default mode network and self-referential processes in depression. Proc Natl Acad Sci USA.

[10] Berman MG, Peltier S, Nee DE, Kross E, Deldin PJ, Jonides J (2011). Depression, rumination and the default network. Soc Cogn Affect Neurosci.

[11] Grimm S, Ernst J, Boesiger P, Schuepbach D, Hell D, Boeker H (2009). Increased self-focus in major depressive disorder is related to neural abnormalities in subcortical-cortical midline structures. Hum Brain Mapp..

[12] Hamilton JP, Farmer M, Fogelman P, Gotlib IH (2015). Depressive rumination, the default-mode network, and the dark matter of clinical neuroscience. Biol Psychiatry.

[13] Greicius MD, Krasnow B, Reiss AL, Menon V (2003). Functional connectivity in the resting brain: a network analysis of the default mode hypothesis. Proc Natl Acad Sci USA.

[14] Greicius MD, Flores BH, Menon V, Glover GH, Solvason HB, Kenna H (2007). Resting-state functional connectivity in major depression: abnormally increased contributions from subgenual cingulate cortex and thalamus. Biol Psychiatry.

[15] Yan CG, Chen X, Li L, Castellanos FX, Bai TJ, Bo QJ (2019). Reduced default mode network functional connectivity in patients with recurrent major depressive disorder. Proc Natl Acad Sci USA.

[16] Hahn A, Wadsak W, Windischberger C, Baldinger P, Höflich AS, Losak J (2012). Differential modulation of the default mode network via serotonin-1A receptors. Proc Natl Acad Sci USA.

[17] Northoff G, Walter M, Schulte RF, Beck J, Dydak U, Henning A (2007). GABA concentrations in the human anterior cingulate cortex predict negative BOLD responses in fMRI. Nat Neurosci..

[18] Conio B, Martino M, Magioncalda P, Escelsior A, Inglese M, Amore M (2020). Opposite effects of dopamine and serotonin on resting-state networks: review and implications for psychiatric disorders. Mol Psychiatry.

[19] Duman RS, Sanacora G, Krystal JH (2019). Altered connectivity in depression: GABA and glutamate neurotransmitter deficits and reversal by novel treatments. Neuron.

[20] Fakhoury M (2016). Revisiting the serotonin hypothesis: implications for major depressive disorders. Mol Neurobiol..

[21] Hirschfeld RM (2000). History and evolution of the monoamine hypothesis of depression. J Clin Psychiatry.

[22] Saulin A, Savli M, Lanzenberger R (2012). Serotonin and molecular neuroimaging in humans using PET. Amino Acids.

[23] Kunisato Y, Okamoto Y, Okada G, Aoyama S, Demoto Y, Munakata A (2011). Modulation of default-mode network activity by acute tryptophan depletion is associated with mood change: a resting state functional magnetic resonance imaging study. Neurosci Res..

[24] Biskup CS, Helmbold K, Baurmann D, Klasen M, Gaber TJ, Bubenzer-Busch S (2016). Resting state default mode network connectivity in children and adolescents with ADHD after acute tryptophan depletion. Acta Psychiatr Scand.

[25] Helmbold K, Zvyagintsev M, Dahmen B, Biskup CS, Bubenzer-Busch S, Gaber TJ (2016). Serotonergic modulation of resting state default mode network connectivity in healthy women. Amino Acids.

[26] Klaassens BL, van Gorsel HC, Khalili-Mahani N, van der Grond J, Wyman BT, Whitcher B (2015). Single-dose serotonergic stimulation shows widespread effects on functional brain connectivity. Neuroimage.

[27] van de Ven V, Wingen M, Kuypers KP, Ramaekers JG, Formisano E (2013). Escitalopram decreases cross-regional functional connectivity within the default-mode network. PLoS ONE.

[28] Schrantee A, Lucassen PJ, Booij J, Reneman L (2018). Serotonin transporter occupancy by the SSRI citalopram predicts default-mode network connectivity. Eur Neuropsychopharmacol.

[29] Buckner RL, Andrews-Hanna JR, Schacter DL (2008). The brain’s default network: anatomy, function, and relevance to disease. Ann N Y. Acad Sci.

[30] Cheng Y, Xu J, Arnone D, Nie B, Yu H, Jiang H (2017). Resting-state brain alteration after a single dose of SSRI administration predicts 8-week remission of patients with major depressive disorder. Psychol Med..

[31] Cullen KR, Klimes-Dougan B, Vu DP, Westlund Schreiner M, Mueller BA, Eberly LE (2016). Neural correlates of antidepressant treatment response in adolescents with major depressive disorder. J Child Adol Psychop.

[32] Li B, Liu L, Friston KJ, Shen H, Wang L, Zeng L (2013). A treatment-resistant default mode subnetwork in major depression. Biol Psychiatr..

[33] Wagner G, de la Cruz F, Köhler S, Bär K (2017). Treatment associated changes of functional connectivity of midbrain/brainstem nuclei in major depressive disorder. Sci. Rep.

[34] Wang L, An J, Gao HM, Zhang P, Chen C, Li K (2019). Duloxetine effects on striatal resting‐state functional connectivity in patients with major depressive disorder. Hum Brain Mapp..

[35] Li L, Li B, Bai Y, Liu W, Wang H, Leung H (2017). Abnormal resting state effective connectivity within the default mode network in major depressive disorder: a spectral dynamic causal modeling study. Brain Behav.

[36] Sheehan DV, Lecrubier Y, Sheehan KH, Amorim P, Janavs J, Weiller E (1998). The mini-international neuropsychiatric interview (M.I.N.I.): the development and validation of a structured diagnostic psychiatric interview for DSM-IV and ICD-10. J Clin Psychiatry.

[37] Rush AJ, Trivedi MH, Ibrahim HM, Carmody TJ, Arnow B, Klein DN (2003). The 16-Item Quick Inventory of Depressive Symptomatology (QIDS), Clinician Rating (QIDS-C), and Self-Report (QIDS-SR): a psychometric evaluation in patients with chronic major depression. Biol Psychiatry.

[38] Hamilton M (1960). A rating scale for depression. J Neurol Neurosurg Psychiatry.

[39] Kennedy SH, Andersen HF, Thase ME (2009). Escitalopram in the treatment of major depressive disorder: a meta-analysis. Curr Med Res Opin..

[40] Cipriani A, Furukawa TA, Salanti G, Chaimani A, Atkinson LZ, Ogawa Y (2018). Comparative efficacy and acceptability of 21 antidepressant drugs for the acute treatment of adults with major depressive disorder: a systematic review and network meta-analysis. Lancet.

[41] Chon MW, Lee J, Chung S, Kim Y, Kim HW (2017). Prescription pattern of antidepressants for children and adolescents in Korea based on nationwide data. J Korean Med Sci..

[42] Tripathi A, Avasthi A, Desousa A, Bhagabati D, Shah N, Kallivayalil RA (2016). Prescription pattern of antidepressants in five tertiary care psychiatric centres of India. Indian J Med Res..

[43] Kennedy SH, Lam RW, McIntyre RS, Tourjman SV, Bhat V, Blier P (2016). Canadian Network for Mood and Anxiety Treatments (CANMAT) 2016 clinical guidelines for the management of adults with major depressive disorder: section 3. Pharmacological treatments. Can J Psychiatry.

[44] Kroenke K, Spitzer RL, Williams JB (2001). The PHQ-9: validity of a brief depression severity measure. J Gen Intern Med..

[45] Chao-Gan Y, Yu-Feng Z (2010). DPARSF: a MATLAB Toolbox for "Pipeline" data analysis of resting-state fMRI. Front Syst Neurosci.

[46] Tanabe J, Miller D, Tregellas J, Freedman R, Meyer FG (2002). Comparison of detrending methods for optimal fMRI preprocessing. Neuroimage.

[47] Yan CG, Craddock RC, Zuo XN, Zang YF, Milham MP (2013). Standardizing the intrinsic brain: towards robust measurement of inter-individual variation in 1000 functional connectomes. Neuroimage.

[48] Nalci A, Rao BD, Liu TT (2017). Global signal regression acts as a temporal downweighting process in resting-state fMRI. Neuroimage.

[49] Gotts SJ, Saad ZS, Jo HJ, Wallace GL, Cox RW, Martin A (2013). The perils of global signal regression for group comparisons: a case study of autism spectrum disorders. Front Hum Neurosci..

[50] Abbott AE, Nair A, Keown CL, Datko M, Jahedi A, Fishman I (2016). Patterns of atypical functional connectivity and behavioral links in autism differ between default, salience, and executive networks. Cereb Cortex.

[51] Power JD, Barnes KA, Snyder AZ, Schlaggar BL, Petersen SE (2012). Spurious but systematic correlations in functional connectivity MRI Networks arise from subject motion. Neuroimage.

[52] Yan CG, Cheung B, Kelly C, Colcombe S, Craddock RC, Di Martino A (2013). A comprehensive assessment of regional variation in the impact of head micromovements on functional connectomics. Neuroimage.

[53] Yeo BT, Krienen FM, Eickhoff SB, Yaakub SN, Fox PT, Buckner RL (2015). Functional specialization and flexibility in human association cortex. Cereb Cortex.

[54] Yeo BT, Krienen FM, Sepulcre J, Sabuncu MR, Lashkari D, Hollinshead M (2011). The organization of the human cerebral cortex estimated by intrinsic functional connectivity. J Neurophysiol..

[55] Dixon ML, Andrews-Hanna JR, Spreng RN, Irving ZC, Mills C, Girn M (2017). Interactions between the default network and dorsal attention network vary across default subsystems, time, and cognitive states. Neuroimage.

[56] Zhu X, Zhu Q, Shen H, Liao W, Yuan F (2017). Rumination and default mode network subsystems connectivity in first-episode, drug-naive young patients with major depressive disorder. Sci Rep.

[57] Gu S, Satterthwaite TD, Medaglia JD, Yang M, Gur RE, Gur RC (2015). Emergence of system roles in normative neurodevelopment. Proc Natl Acad Sci USA.

[58] Zalesky A, Fornito A, Bullmore ET (2010). Network-based statistic: identifying differences in brain networks. Neuroimage.

[59] Power JD, Cohen AL, Nelson SM, Wig GS, Barnes KA, Church JA (2011). Functional network organization of the human brain. Neuron.

[60] Chen X, Chen NX, Shen YQ, Li HX, Li L, Lu B (2020). The subsystem mechanism of default mode network underlying rumination: a reproducible neuroimaging study. Neuroimage.

[61] Price RB, Lane S, Gates K, Kraynak TE, Horner MS, Thase ME (2017). Parsing heterogeneity in the brain connectivity of depressed and healthy adults during positive mood. Biol Psychiatry.

[62] Price RB, Gates K, Kraynak TE, Thase ME, Siegle GJ (2017). Data-driven subgroups in depression derived from directed functional connectivity paths at rest. Neuropsychopharmacology.

[63] Drysdale AT, Grosenick L, Downar J, Dunlop K, Mansouri F, Meng Y (2017). Resting-state connectivity biomarkers define neurophysiological subtypes of depression. Nat Med..

[64] Liang S, Deng W, Li X, Greenshaw AJ, Wang Q, Li M (2020). Biotypes of major depressive disorder: neuroimaging evidence from resting-state default mode network patterns. Neuroimage Clin.

[65] Cullen KR, Gee DG, Klimes-Dougan B, Gabbay V, Hulvershorn L, Mueller BA (2009). A preliminary study of functional connectivity in comorbid adolescent depression. Neurosci Lett..

[66] Lui S, Wu Q, Qiu L, Yang X, Kuang W, Chan RC (2011). Resting-state functional connectivity in treatment-resistant depression. Am J Psychiatry.

[67] van Tol MJ, Li M, Metzger CD, Hailla N, Horn DI, Li W (2014). Local cortical thinning links to resting-state disconnectivity in major depressive disorder. Psychol Med..

[68] Peng D, Shi F, Shen T, Peng Z, Zhang C, Liu X (2014). Altered brain network modules induce helplessness in major depressive disorder. J Affect Disord.

[69] Chen Y, Wang C, Zhu X, Tan Y, Zhong Y (2015). Aberrant connectivity within the default mode network in first-episode, treatment-naïve major depressive disorder. J Affect Disord.

[70] Sawaya H, Johnson K, Schmidt M, Arana A, Chahine G, Atoui M (2015). Resting-state functional connectivity of antero-medial prefrontal cortex sub-regions in major depression and relationship to emotional intelligence. Int J Neuropsychopharmacol.

[71] Schilbach L, Hoffstaedter F, Müller V, Cieslik EC, Goya-Maldonado R, Trost S (2016). Transdiagnostic commonalities and differences in resting state functional connectivity of the default mode network in schizophrenia and major depression. Neuroimage Clin.

[72] Yang XH, Tian K, Wang DF, Wang Y, Cheung E, Xie GR (2017). Anhedonia correlates with abnormal functional connectivity of the superior temporal gyrus and the caudate nucleus in patients with first-episode drug-naive major depressive disorder. J Affect Disord.

[73] Andreescu C, Tudorascu DL, Butters MA, Tamburo E, Patel M, Price J (2013). Resting state functional connectivity and treatment response in late-life depression. Psychiatry Res.

[74] Arnone D, Wise T, Walker C, Cowen PJ, Howes O, Selvaraj S (2018). The effects of serotonin modulation on medial prefrontal connectivity strength and stability: a pharmacological fMRI study with citalopram. Prog Neuropsychopharmacol Biol Psychiatry.

[75] Barbas H, Ghashghaei H, Dombrowski SM, Rempel-Clower NL (1999). Medial prefrontal cortices are unified by common connections with superior temporal cortices and distinguished by input from memory-related areas in the rhesus monkey. J Comp Neurol..

[76] Andrews-Hanna JR, Smallwood J, Spreng RN (2014). The default network and self-generated thought: component processes, dynamic control, and clinical relevance. Ann N Y Acad Sci.

[77] Andrews-Hanna JR (2012). The brain’s default network and its adaptive role in internal mentation. Neuroscientist.

[78] Young KD, Siegle GJ, Bodurka J, Drevets WC (2016). Amygdala activity during autobiographical memory recall in depressed and vulnerable individuals: association with symptom severity and autobiographical overgenerality. Am J Psychiatry.

[79] Kim D, Yoon KL (2020). Emotional response to autobiographical memories in depression: less happiness to positive and more sadness to negative memories. Cogn Behav Ther.

[80] Young KD, Friedman ES, Collier A, Berman SR, Feldmiller J, Haggerty AE (2020). Response to SSRI intervention and amygdala activity during self-referential processing in major depressive disorder. Neuroimage Clin.

[81] Poeppl TB, Müller VI, Hoffstaedter F, Bzdok D, Laird AR, Fox PT (2016). Imbalance in subregional connectivity of the right temporoparietal junction in major depression. Hum Brain Mapp..

[82] Hasler G, van der Veen JW, Tumonis T, Meyers N, Shen J, Drevets WC (2007). Reduced prefrontal glutamate/glutamine and gamma-aminobutyric acid levels in major depression determined using proton magnetic resonance spectroscopy. Arch Gen Psychiatry.

[83] Craddock RC, James GA, Holtzheimer PR, Hu XP, Mayberg HS (2012). A whole brain fMRI atlas generated via spatially constrained spectral clustering. Hum Brain Mapp..

[84] Cao H, Plichta MM, Schäfer A, Haddad L, Grimm O, Schneider M (2014). Test-retest reliability of fMRI-based graph theoretical properties during working memory, emotion processing, and resting state. Neuroimage.

[85] An J, Li L, Wang L, Su YA, Wang Y, Li K (2019). Striatal functional connectivity alterations after two-week antidepressant treatment associated to enduring clinical improvement in major depressive disorder. Front Psychiatry.

[86] Wang L, Li K, Zhang Q, Zeng Y, Dai W, Su Y (2014). Short-term effects of escitalopram on regional brain function in first-episode drug-naive patients with major depressive disorder assessed by resting-state functional magnetic resonance imaging. Psychol Med.

[87] Tadayonnejad R, Ajilore O, Mickey BJ, Crane NA, Hsu DT, Kumar A (2016). Pharmacological modulation of pulvinar resting-state regional oscillations and network dynamics in major depression. Psychiatry Res.

[88] Karim HT, Andreescu C, Tudorascu D, Smagula SF, Butters MA, Karp JF (2017). Intrinsic functional connectivity in late-life depression: trajectories over the course of pharmacotherapy in remitters and non-remitters. Mol Psychiatry.

[89] Fu CH, Costafreda SG, Sankar A, Adams TM, Rasenick MM, Liu P (2015). Multimodal functional and structural neuroimaging investigation of major depressive disorder following treatment with duloxetine. BMC Psychiatry.

[90] Anand A, Li Y, Wang Y, Wu J, Gao S, Bukhari L (2005). Antidepressant effect on connectivity of the mood-regulating circuit: an fMRI study. Neuropsychopharmacology.

[91] Yang R, Zhang H, Wu X, Yang J, Ma M, Gao Y (2014). Hypothalamus-anchored resting brain network changes before and after sertraline treatment in major depression. Biomed Res Int..

[92] Lebois L, Li M, Baker JT, Wolff JD, Wang D, Lambros AM (2021). Large-scale functional brain network architecture changes associated with trauma-related dissociation. Am J Psychiatry.

[93] Wang D, Buckner RL, Fox MD, Holt DJ, Holmes AJ, Stoecklein S (2015). Parcellating cortical functional networks in individuals. Nat Neurosci..

[94] Diaz BA, Van Der Sluis S, Moens S, Benjamins JS, Migliorati F, Stoffers D (2013). The Amsterdam resting-state questionnaire reveals multiple phenotypes of resting-state cognition. Front Hum Neurosci..

