# Supplementary material for: Effects of escitalopram therapy on resting-state functional connectivity of subsystems of the default mode network in unmedicated patients with major depressive disorder
Source: Transl Psychiatry. 2021 Dec 13;11:634. doi: 10.1038/s41398-021-01754-4 (PMC8668990; doi:10.1038/s41398-021-01754-4)
Supplement: Supplementary file 1 — SUPPLEMENTAL MATERIAL [file 41398_2021_1754_MOESM1_ESM.docx]

**Supplementary materials**

**Confirmation analysis of DMN regions using Power 264 Atlas**

In order to exclude the confounding factor from ROI locations, we repeated our analyses using the DMN regions from the Power 264 atlas as the ROIs. For the 58 ROIs of DMN in the Power atlas^1^, we intersected them with Yeo’s DMN template^2,3^ to exclude 11 ROIs, which resulted in 47 ROIs for the subsequent analyses. Among 47 ROIs, 21 ROIs were contained within the core subsystem and 20 ROIs were contained within the dMPFC subsystem, while 6 ROIs were contained within the MTL subsystem. Functional connectivity between all pairs of the 47 ROIs resulted in a 47×47 connectivity matrix for each participant. Subsequently, within-subsystem and between-subsystem connectivity for the 3 subsystems were computed on connectivity matrices using the same methods for the main analyses.

**References:**

1. Power, J. D., Cohen, A. L., Nelson, S. M., Wig, G. S., Barnes, K. A.,Church, J. A. et al., Functional Network Organization of the Human Brain. *Neuron* **72**, 665-678 (2011).

2. Dixon, M. L., Andrews-Hanna, J. R., Spreng, R. N., Irving, Z. C., Mills, C.,Girn, M. et al., Interactions Between the Default Network and Dorsal Attention Network Vary Across Default Subsystems, Time, and Cognitive States. *Neuroimage* **147**, 632-649 (2017).

3. Yeo, B. T., Krienen, F. M., Sepulcre, J., Sabuncu, M. R., Lashkari, D.,Hollinshead, M. et al., The Organization of the Human Cerebral Cortex Estimated by Intrinsic Functional Connectivity. *J. Neurophysiol.* **106**, 1125-1165 (2011).

TABLE S1. ROIs used in the present study

| **ROI Index** | **Hemisphere** | **Network Name** | **Full Component Name** | **Abbreviation** |
| --- | --- | --- | --- | --- |
| 1 | LH | Default_core | inferior parietal lobule | IPL |
| 2 | LH | Default_core | dorsal prefrontal cortex | PFCd |
| 3 | LH | Default_core | precuneus posterior cingulate cortex | pCunPCC |
| 4 | LH | Default_core | medial prefrontal cortex | PFCm |
| 5 | RH | Default_core | temporal | Temp |
| 6 | RH | Default_core | inferior parietal lobule | IPL |
| 7 | RH | Default_core | dorsal prefrontal cortex | PFCd |
| 8 | RH | Default_core | precuneus posterior cingulate cortex | pCunPCC |
| 9 | RH | Default_core | medial prefrontal cortex | PFCm |
| 10 | LH | Default_dMPFC | temporal | Temp |
| 11 | LH | Default_dMPFC | inferior parietal lobule | IPL |
| 12 | LH | Default_dMPFC | dorsal prefrontal cortex | PFCd |
| 13 | LH | Default_dMPFC | lateral prefrontal cortex | PFCl |
| 14 | LH | Default_dMPFC | ventral prefrontal cortex | PFCv |
| 15 | RH | Default_dMPFC | temporal | Temp |
| 16 | RH | Default_dMPFC | anterior temporal | AntTemp |
| 17 | RH | Default_dMPFC | dorsal prefrontal cortex | PFCd |
| 18 | RH | Default_dMPFC | ventral prefrontal cortex | PFCv |
| 19 | LH | Default_MTL | inferior parietal lobule | IPL |
| 20 | LH | Default_MTL | retrosplenial | Rsp |
| 21 | LH | Default_MTL | parahippocampal cortex | PHC |
| 22 | RH | Default_MTL | inferior parietal lobule | IPL |
| 23 | RH | Default_MTL | retrosplenial | Rsp |
| 24 | RH | Default_MTL | parahippocampal cortex | PHC |

Table S2. Main effects and interaction effect on the rsFC (z transformed) within and between the DMN subsystems for responders

and non-responders

|  |  | MDD responders baseline  (mean ± SD) | MDD responders week 12 (mean ± SD) | MDD Non- responders baseline  (mean ± SD) | MDD Non- responders week 12  (mean ± SD) | F group | F time | F interaction |
| --- | --- | --- | --- | --- | --- | --- | --- | --- |
|  |  |  |  |  |  | (P) | (P) | (P) |
|  |  |  |  |  |  |  |  |  |
| Within | Core subsystem | 0.56 ± 0.15 | 0.64 ± 0.16 | 0.57 ± 0.16 | 0.68 ± 0.10 | 0.25(0.62) | 6.29(0.051) | 0.32(0.66) |
| subsystem | dMPFC subsystem | 0.55 ± 0.13 | 0.62 ± 0.15 | 0.63 ± 0.12 | 0.66 ± 0.13 | 0.55(0.62) | 1.93(0.26) | 0.19(0.66) |
|  | MTL subsystem | 0.46 ± 0.14 | 0.49 ± 0.16 | 0.57 ± 0.11 | 0.51 ± 0.18 | 0.25(0.62) | 0.13(0.72) | 1.32(0.66) |
|  |  |  |  |  |  |  |  |  |
| Between | Core-dMPFC subsystem | 0.34 ± 0.13 | 0.39 ± 0.17 | 0.37 ± 0.11 | 0.48 ± 0.09 | 1.18(0.86) | 6.43(0.048*) | 0.83(0.64) |
| subsystem | Core-MTL subsystem | 0.24 ± 0.13 | 0.26 ± 0.15 | 0.25 ± 0.11 | 0.27 ± 0.12 | 0.01(0.95) | 0.54(0.47) | 0.04(0.85) |
|  | dMPFC-MTL subsystem | 0.05 ± 0.12 | 0.06 ± 0.14 | 0.06 ± 0.11 | 0.12 ± 0.13 | 0.09(0.95) | 1.14(0.44) | 0.65(0.64) |

Notes: P values after FDR correction. *significant after FDR correction (*P* < 0.05). Abbreviations: MDD: Major depressive disorder; dMPFC: dorsal medial prefrontal cortex; MTL: medial temporal lobe.

Table S3. Paired t-test results for the rsFC (z transformed) within and between the DMN subsystems between at baseline and after the 12-week interval in the healthy controls

|  |  | HCs baseline (mean ± SD) | HCs week 12 (mean ± SD) | t (P) |
| --- | --- | --- | --- | --- |
|  |  |  |  |  |
|  |  |  |  |  |
| Within | Core subsystem | 0.68 ± 0.18 | 0.66 ± 0.17 | 0.56(0.58) |
| subsystem | dMPFC subsystem | 0.67 ± 0.17 | 0.68 ± 0.13 | 0.26(0.79) |
|  | MTL subsystem | 0.53 ± 0.15 | 0.57 ± 0.20 | 1.83(0.07) |
|  |  |  |  |  |
| Between | Core-dMPFC subsystem | 0.43 ± 0.15 | 0.42 ± 0.11 | 0.42(0.67) |
| subsystem | Core-MTL subsystem | 0.28 ± 0.18 | 0.30 ± 0.16 | 0.99(0.33) |
|  | dMPFC-MTL subsystem | 0.05 ± 0.16 | 0.06 ± 0.14 | 0.23(0.82) |

Notes: P values after FDR correction. Abbreviations: HC: Healthy controls; Major depressive disorder; dMPFC: dorsal medial prefrontal cortex; MTL: medial temporal lobe.


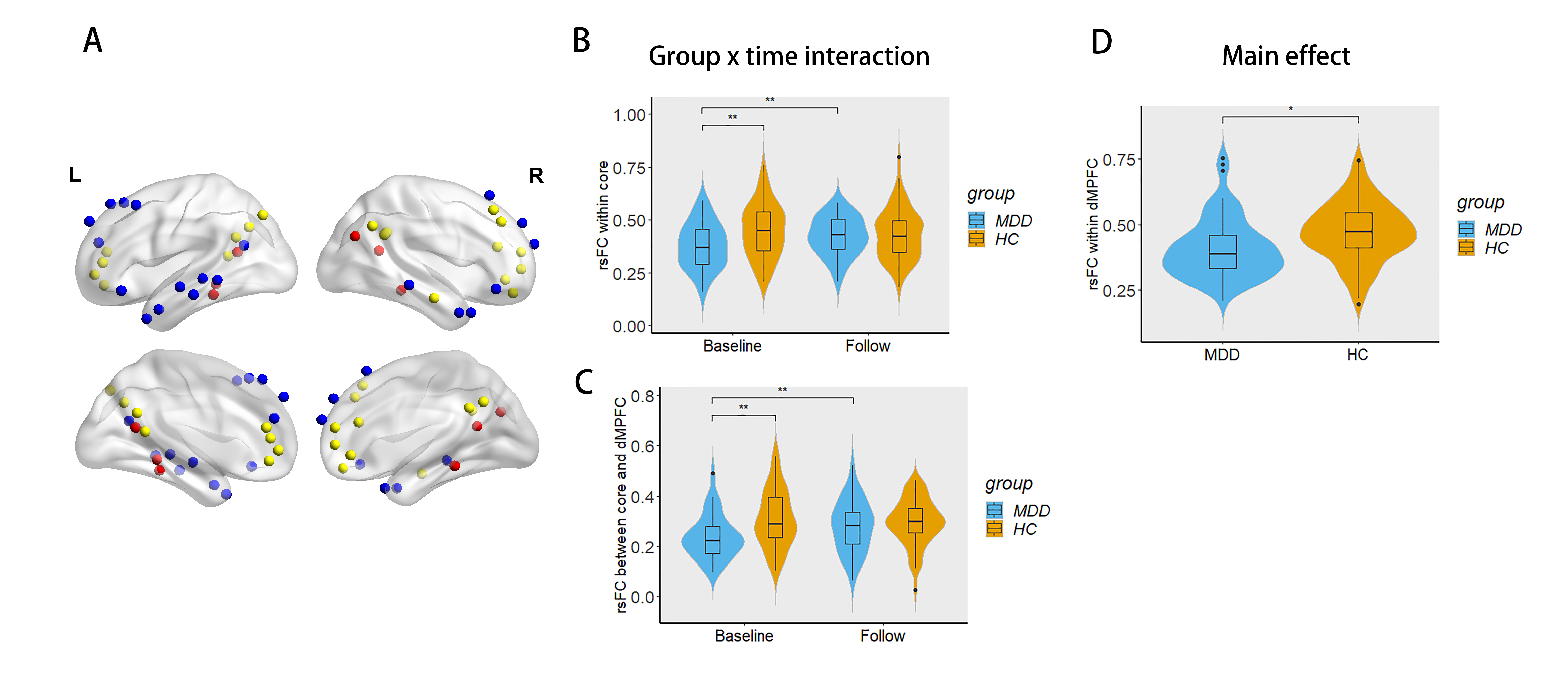


FIGURE S1. The interaction and main effect in the rsFC within and between the DMN subsystems obtained by using the DMN regions from the Power Atlas. Panel A shows spatial distributions of the DMN regions in the Power Atlas^1^. Panel B shows the significant interaction effect on the within-subsystem rsFC of the core subsystem and the between-subsystem rsFC of the core and dMPFC subsystem using violin plots. Panel C shows the significant group main effect on the within-subsystem rsFC in the dMPFC subsystems using violin plots.

***P* < 0.01; **P* < 0.05 for post-hoc analyses.


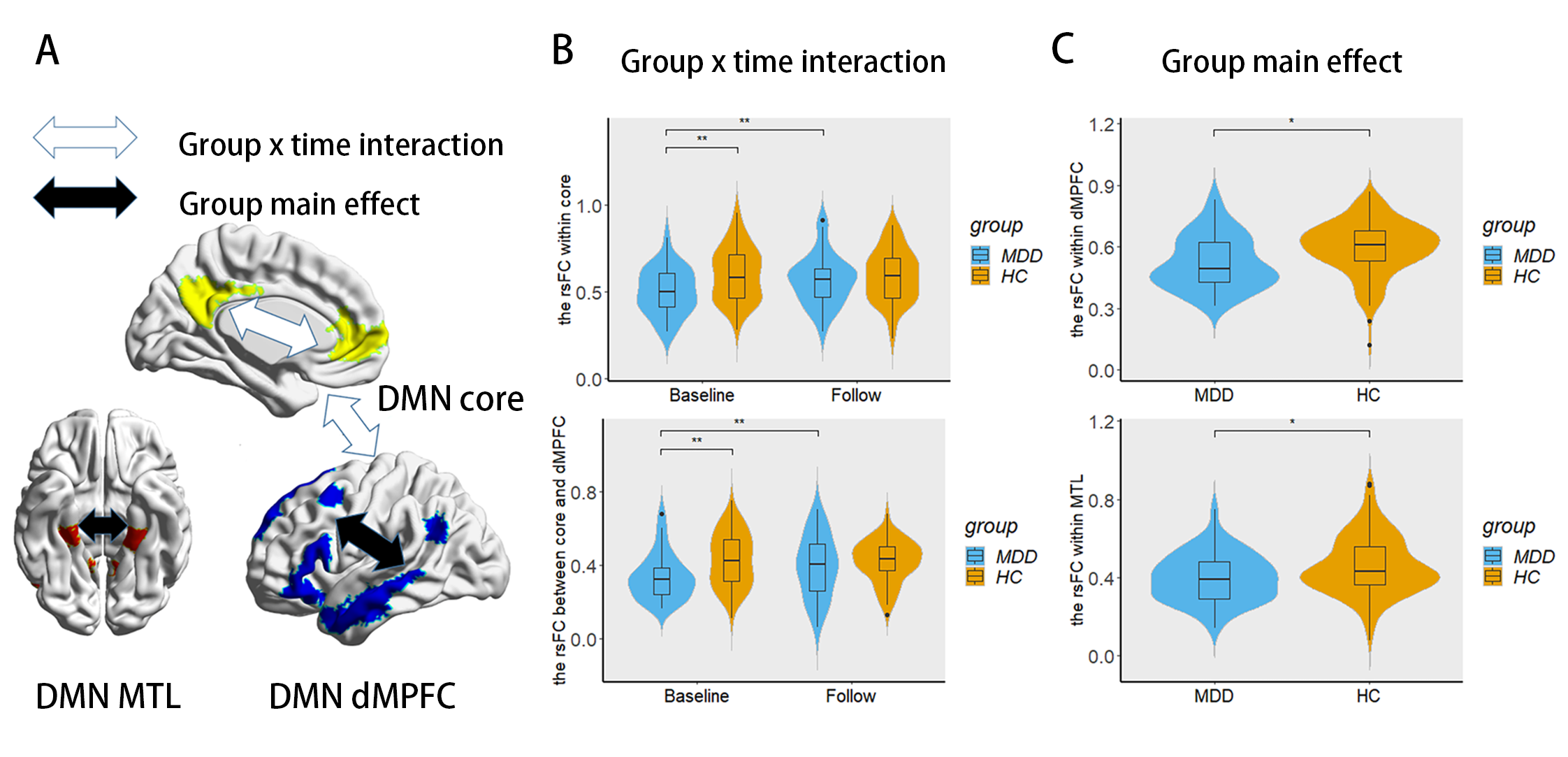


FIGURE S2. The interaction effect and main effect on the rsFC within and between the DMN subsystems while comparing the MDD responders with the healthy controls. Panel A summarizes the interaction effect and main effect within and between the DMN subsystems. Panel B shows the interaction effect on the within-subsystem rsFC of the core subsystem and the between-subsystem rsFC of the core and dMPFC subsystem using violin plots. Panel C shows the group main effect on the within-subsystem rsFC in the dMPFC and MTL subsystems using violin plots.

***P* < 0.01; **P* < 0.05 for post-hoc analyses.
